# Supplementary material for: Theoretical modelling of wakes from retractable flapping wings in forward flight
Source: PeerJ. 2013 Jul 16;1:e105. doi: 10.7717/peerj.105 (PMC3719125; doi:10.7717/peerj.105)
Supplement: Supplemental Information 1 — nt = number of timesteps per cycle nf = number of filaments per wing nm = number of markers per cycle. [file peerj-01-105-s001.docx]

# Supplementary Material for Visualising Wakes from Lifting Surfaces Figure 4

Table showing values of normalised marker position, *p_norm_*:

|  |  | *n_t_* = 50 | *n_t_* = 100 | *n_t_* = 200 | *n_t_* = 400 | *n_t_* = 800 |
| --- | --- | --- | --- | --- | --- | --- |
| *n_f_* = 4 | *n_m_* = 20 | 0.9134 | 0.5855 | 0.5829 | 0.5693 | 0.5650 |
|  | *n_m_* = 30 | 0.5882 | 0.5776 | 0.5782 | 0.5756 | 0.5771 |
| *n_f_* = 8 | *n_m_* = 20 | 0.6046 | 0.5863 | 0.5832 | 0.5724 | 0.5678 |
|  | *n_m_* = 30 | 0.5870 | 0.5799 | 0.5798 | 0.5748 | 0.5785 |

*n_t_* = number of timesteps per cycle

*n_f_* = number of filaments per wing

*n_m_* = number of markers per cycle
